# Cognitive functioning in opioid-dependent patients treated with buprenorphine, methadone, and other psychoactive medications: stability and correlates

**DOI:** 10.1186/1472-6904-11-13

**Published:** 2011-08-21

**Authors:** Pekka Rapeli, Carola Fabritius, Hely Kalska, Hannu Alho

**Affiliations:** 1 Department of Psychiatry. Helsinki University Central Hospital, Finland; 2 Department of Mental Health and Substance Abuse Services, National Institute for Health and Welfare (THL), Finland; 3 Institute of Behavioural Sciences, University of Helsinki, Finland; 4 Research Unit of Substance Abuse Medicine, University of Helsinki, Finland

## Abstract

**Background:**

In many but not in all neuropsychological studies buprenorphine-treated opioid-dependent patients have shown fewer cognitive deficits than patients treated with methadone. In order to examine if hypothesized cognitive advantage of buprenorphine in relation to methadone is seen in clinical patients we did a neuropsychological follow-up study in unselected sample of buprenorphine- vs. methadone-treated patients.

**Methods:**

In part I of the study fourteen buprenorphine-treated and 12 methadone-treated patients were tested by cognitive tests within two months (T1), 6-9 months (T2), and 12 - 17 months (T3) from the start of opioid substitution treatment. Fourteen healthy controls were examined at similar intervals. Benzodiazepine and other psychoactive comedications were common among the patients. Test results were analyzed with repeated measures analysis of variance and planned contrasts. In part II of the study the patient sample was extended to include 36 patients at T2 and T3. Correlations between cognitive functioning and medication, substance abuse, or demographic variables were then analyzed.

**Results:**

In part I methadone patients were inferior to healthy controls tests in all tests measuring attention, working memory, or verbal memory. Buprenorphine patients were inferior to healthy controls in the first working memory task, the Paced Auditory Serial Addition Task and verbal memory. In the second working memory task, the Letter-Number Sequencing, their performance improved between T2 and T3. In part II only group membership (buprenorphine vs. methadone) correlated significantly with attention performance and improvement in the Letter-Number Sequencing. High frequency of substance abuse in the past month was associated with poor performance in the Letter-Number Sequencing.

**Conclusions:**

The results underline the differences between non-randomized and randomized studies comparing cognitive performance in opioid substitution treated patients (fewer deficits in buprenorphine patients vs. no difference between buprenorphine and methadone patients, respectively). Possible reasons for this are discussed.

## Background

Opioid agonists buprenorphine and methadone prevent opioid withdrawal symptoms and reduce craving for opioids [1,2]. Both drugs are used in opioid substitution treatment (OST), also known as opioid maintenance treatment. OST has proven effective in reducing illicit drug use, somatic diseases, mortality, and social or mental health problems in opioid-dependent patients [3,4]. Cognitive effects of OST drugs have been examined in clinical and experimental studies, but the results have been mixed. Studies comparing OST patients against healthy controls have, in general, shown cognitive impairment among patients [5-8]. Yet, it has not been proven that the impairment would be specifically related to opioid substitution drugs [5,9,10]. In non-randomized studies, however, buprenorphine-treated opioid-dependent patients have performed better than methadone patients in several cognitive tests [7,11-13].

It is important to know if the possible cognitive differences between unselected buprenorphine vs. methadone patients are stabile during the treatment and what are the correlates of cognitive performance. Therefore, we compared cognitive performance of buprenorphine and methadone patients against healthy controls thrice (T1 - T3) during the first year in the OST by (part I of the study). In part II we analyzed correlates of cognitive performance in patients after six (T2) and twelve (T3) months in treatment by using extended patient pool. The present study is an extension to our previous studies [7,14].

### Part I: Stability

Opioid and dopamine systems in the brain have important interactions, and current opioid drug use may negatively affect cognitive functioning, especially working memory [15-17]. However, Pirastu et al. have presented evidence that buprenorphine as being a partial mu opioid agonist and kappa opioid receptor antagonist may improve cognitive performance after long-term opioid abuse. According to them methadone as being a full mu opioid agonist may lack properties for supporting normal cognitive function [18]. Also, there is evidence that adverse interactive effects benzodiazepines (BZD) and opioid substitution drugs on cognitive performance are greater for methadone than buprenorphine [19,20]. Therefore, we hypothesized that patients treated with buprenorphine combined in most cases with BZD and other comedications would show greater cognitive improvement in long-term treatment in comparison to methadone-treated ones.

### Part II: correlates

In the part II of the study the patient sample was extended to include additional patients examined at all test points, but whose data were excluded at T1. After this, data from 36 patients could be analyzed at T2 and T3. We hypothesized that there would be negative correlations between medication variables (opioid agonist dose, BZD dose, and the number of psychoactive drugs) and cognitive performance in opioid-dependent patients treated either with buprenorphine or methadone. In addition, we hypothesized that those with the highest opioid dose would have higher BZD doses, because BZDs have been associated with craving for higher opioid dose [21]. The negative effects of methadone and buprenorphine on cognition are dose-dependent in healthy volunteers, although little is known about the development of tolerance [22,23]. It is known that BZDs have negative effects on memory performance in opioid substitution treated patients, and these effects are stronger for methadone than for buprenorphine [24]. Little is known about possible effects of polypharmacy on cognition in opioid-agonist treated patients. However, in other patient populations, those patients treated with several drugs perform worse in cognitive tests than patients treated with single drug [25-27].

Negative correlations were also hypothesized between cognitive performance and frequency of substance abuse in the past month, benzodiazepine dosage, the number of other psychoactive drugs, early onset of substance abuse, early-onset mental health or behavioral problems, opioid-related overdoses, and duration of lifetime alcohol abuse. In our sample recent alcohol and/or cannabis abuse were common, and these negatively affect cognitive function [28-31]. Early onset substance abuse and childhood mental health or behavioral problems have been associated with poor adult cognitive functioning among individuals with substance abuse problems [32-35]. High number of opioid-related overdoses, lifetime alcohol abuse, and low level of education have all been associated with poor cognitive performance among opioid-dependent patients [5,36,37]. Verbal intelligence (IQ) and years of education were hypothesized to correlate positively with memory performance.

## Methods

All participants included in the study were between 18 - 50 years of age and participated voluntarily. Inclusion criteria for patients were opioid dependence and BZD dependence or abuse according to Diagnostic and Statistical Manual of Mental Disorders (DSM-IV), treatment of opioid dependence with methadone, buprenorphine, or buprenorphine/naloxone. We excluded participants with uncontrolled polysubstance abuse, acute alcohol abuse, or acute axis I psychiatric morbidity according to DSM-IV other than substance abuse disorders. Full description of our inclusion and exclusion criteria is given in our previous report [7].

In order to screen for substance abuse an urine sample was collected from each patient on each day of testing and at least once in the preceding week. Each healthy control participant was screened for substance abuse once during the study period. In addition, we interviewed all participants about their past month and lifetime substance use by using the European Addiction Severity Index as a basis for further inquiry [38]. If any indication of intoxication was observed, we excluded them. Breath alcohol testing was used when considered necessary. Participants who had used within 24 h alcohol more than four/five drinks (females/males, respectively) or significant as-needed benzodiazepine dose (5 mg or more as diazepam equivalent dose) were excluded as well. The study protocol was accepted by the Ethics Committee of Helsinki University Central Hospital. We obtained a written informed consent according to the Declaration of Helsinki from all participants, and paid them € 60 if they attended all study visits.

### Part I participants

Participants who were eligible for T1-T3 follow-up (sample I) represent 42% (14/35) of the all buprenorphine patients tested at T1, 55% (12/22) of the methadone patients and 78% (14/18) of the healthy controls, respectively. To test whether the follow-up completers of either group were significantly different from the non-completers of that group, we compared these groups by independent samples *t-*tests, chi-square tests, or Mann-Whitney *U*-tests (*p*-value = 0.05). No statistically significant differences emerged in demographic, medication or cognitive variables. Because there were few follow-up non-completers (n = 4) among the potential healthy controls, these comparisons were not made in healthy controls.

When the groups were compared on demographic variables with analysis of variance (ANOVA) or chi-square-test (Table 1) there were no statistically significant differences in age, sex, or estimated premorbid intelligence. Healthy controls had completed more years in education than either one of the patient groups. Because BZD use on prescription was very common, their doses were converted to diazepam equivalent doses according to the conversion tables given by Nelson and Chouinard [39]. Temazepam doses were halved in order to account for their use as hypnotics on the night before testing. Substance abuse in the past month was estimated as frequency of use. Because accurate number of the days of abuse was hard to obtain we dichotomized the frequency of the past month substance abuse into two categories. The first category was labeled as low to moderate use, and it included abstinence or substance abuse up to two days a week. The second category was labeled as high frequency group and included all the participants with substance use of three days a week or more. This classification was based on the findings showing that mean three days of substance use a week is one of the threshold values for getting into serious substance abuse problems [40,41]. In the buprenorphine group, 79% of the patients were given buprenorphine/naloxone at all test points. Thus, they were also given sublingual naloxone in the ratio 1:4 combined with their buprenorphine dose. When the tablet is taken sublingually the absorption of naloxone is low and eliminates within first hours [42]. It has been concluded that naloxone has minimal, if any effect, on the bioavailability or pharmacokinetics of buprenorphine [43,44]. Also, buprenorphine and buprenorphine/naloxone have similar physiological effects [43]. On the basis of these findings, we combined patients using either one of the buprenorphine compounds. Table 2 shows medication characteristics of the sample I within the last 24 h before testing. Both patient groups used more psychoactive medications than healthy controls.

**Table 1:** Group demographics in sample I

	Buprenorphine (n = 14)	Methadone (n = 12)	Healthy control (n = 14)	Group comparison *p*-values
Age (*M *± *SD*)	30 ± 7	31 ± 8	29 ± 10	*ns*

Sex (female/male)	36%/64%	50%/50%	50%/50%	*ns*

Intelligence^a ^(*M ± SD*)	101 ± 11	98 ± 9	105 ± 8	*ns*

Education, years	10 ± 2	10 ± 1	13 ± 1	BN & M < HC ***

Main opioid of abuse used within last month at T1 (%)				
Buprenorphine	93%	83%?	-	*ns^b^*
Heroin	7%	17%	-	*ns^b^*

Days in opioid substitution treatment at test (*M ± SD*)				
T1	21 ± 15	20 ± 14	-	*ns*^b^
T2	210 ± 20	200 ± 28	-	*ns*^b^
T3	414 ± 46	405 ± 31	-	*ns*^b^

Examined in inpatient settings %				
T1	21%	25%	-	*ns*^b^
T2	7%	0%	-	*ns*^b^
T3	7%	8%	-	*ns*^b^

Participants with high frequency of use of any substance of abuse ^c ^%				
T1	86%	67%	14%	BN > HC ***; M > HC *
T2	29%	42%	7%	*ns *; *ns*
T3	36%	33%	7%	*ns *; *ns*
	T2 < T1**	T3 < T1*		
	T3 < T1*			

Participants with the past month extra doses of any opioid ^d^, %				
T1	86%	92%	-	*ns*^b^
T2	29%	33%	-	*ns*^b^
T3	36%	33%	-	*ns*^b^
	T2 < T1**	T2 < T1**		
	T3 < T1*	T3 < T1**		

Participants with the past month nicotine use (daily)				
T1	100%	100%	36%	BN & M > HC ***
T2	100%	100%	36%	BN & M > HC ***
T3	100%	93%	29%	BN > HC **; M > HC ***

Note. BN = buprenorphine patients, HC = healthy control group, and M = methadone patients.

^a ^Estimation based on the vocabulary and picture completion subtests of the Wechsler Adult Intelligence Scale - Revised (WAIS-R) [67].

^b ^Tested only between patient groups.

^c ^High frequency = three or more days a week. Alcohol use was taken into account if it was at least mean weekly 16 portions (12 g) for females and 24 portions for males or binge drinking occurred on any day.

^d ^Extra doses of any non-prescribed opioid use during the recent month seen in drugs screens or admitted by the patients.

> = superior than, *** = statistically significant at level *p *< 0.001. ** = statistically significant at level *p *< 0.01. * = statistically significant at level *p *< 0.05.

**Table 2:** Medications given to participants within the last 24 h before testing in sample I

	Buprenorphine (n = 14)	Methadone (n = 12)	Healthy control (n = 14)	Group or time point comparison *p*-values
Opioid agonist drug, dose				
Buprenorphine (*M *± *SD*; (range) )				
T1	16 ± 3 mg (12 - 24 mg)	-	-	-
T2	20 ± 5 mg (14 - 28 mg)	-	-	T2 > T1**
T3 Methadone (*M *± *SD*;(range) )	21 ± 6 mg (6 - 28 mg)	-	-	T3 > T1**
T1	-	71 ± 39 mg (30 - 135 mg)	-	-
T2	-	127 ± 36 mg (80 - 180 mg)	-	T2 > T1 ***
T3	-	135 ± 34 mg (75 - 180 mg)	-	T3 > T1 ***

Participants treated with BZD medication			'	
T1	79%	100%	0%	BN & M > HC ***
BZD dose at T1 (*M *± *SD*)	20 ± 17 mg	21 ± 11 mg	-	*ns *^a^
T2	71%	100%	0%	BN & M > HC ***
BZD dose at T2 (*M *± *SD*)	16 ± 11 mg	22 ± 11 mg	-	*ns *^a^
T3	64%	100%	0%	BN & M > HC ***
BZD dose at T3 (*M *± *SD*)	13 ± 12 mg	22 ± 9. mg	-	BN < M *

Number of other medications with possible cognitive effects ^b^ (*M *± *SD *(range))				
T1	1.9 ± 1.1 (0 - 4)	3.0 ± 1.3 (0 - 5)	0.2 ± 0.4 (0 - 1)	BN & M > HC ***^; ^M > BN *
T2	1.9 ± 1.2 (0 - 3)	2.3 ± 0.8 (1 - 4)	0.2 ± 0.4 (0 -1)	BN & M > HC ***
T3	1.8 ± 1.3 (0 -4)	2.2 ± 1.0 (1 -4)	0.2 ± 0.4 (0 -1)	BN & M > HC ***

^a ^Tested only between patient groups.

^b ^These included antidepressants, neuroleptics (used with anxiolytic indications), non-benzodiazepine hypnotics, and substance abuse withdrawal symptom or (non-opioid) pain relievers. There were no significant differences between time points within the groups in medication variables.

> = superior than, *** = statistically significant at level *p *< 0.001. ** = statistically significant at level *p *< 0.01. * = statistically significant at level *p *< 0.05.

### Part II participants

Sample II (n = 36) included 51% of all the buprenorphine-treated and 59% of methadone-treated patients who entered the follow-up at T1. The methadone group included also five patients who were tested without opioid medication at T1, but who then started methadone treatment within few days after the testing. Thus, all patients were tested after minimum 6 (T2) and 12 months (T3) of OST. They had been tested at start of their treatment, but were excluded from the part I sample. Substance abuse history variables included in the analyses were onset ages of any substance and opioid abuse, years of heavy alcohol use, and the number of self-reported opioid-related overdoses. Whenever possible, the data was checked using medical reports. It turned out that no reliable information about the number of opioid-related overdoses could be obtained. Therefore this variable was excluded from the analyses. Current substance abuse variables were frequency of substance abuse in the past month (low vs. high) and drug screen result (positive vs. negative). Medication drug use variables that were examined included opioid substitution drug (buprenorphine vs. methadone), benzodiazepine dose (diazepam equivalent), and the number of other psychoactive drugs other than opioid substitution drug. Demographic variables included in the analyses were age, sex, years of education, early neurobehavioral problems, and verbal IQ. Data about childhood mental health or behavioral problems was gathered using the Childhood Behavioral Checklist as a basis for interview, and medical reports were used, whenever possible [45]. Those participants who had had treatment or referral to special services due to mental health or behavioral problems before the onset of substance abuse were rated as early-onset neurobehavioral problem group (31%). If significant change was seen in cognitive performance then change (T3 - T2) in that that variable, as well in medication, and substance use changes were analyzed. Medication and substance abuse change variables were made more reliable by dichotomizing the data. Change in opioid drug dose between T2 and T3 was dichotomized as steady or reduced dose group (58%) or higher dose group (42%). Change in BZD dose between T2 and T2 was grouped respectively. The majority of the patients belonged to steady or reduced BZD dose group (83%), and the rest (17%) had higher BZD dose at T3. All those who reduced their frequency of substance abuse as indicated by the shift from the high frequency group to low to moderate frequency group were put into group of reduced substance abuse. This group included also the patients who belonged to the low to moderate frequency group at both time points, totaling 58% of the patients. The rest were put into group of non-reduced substance abuse (42%). Change in the number of psychoactive drugs was dichotomized similarly. All those with less psychoactive drugs at T3 in comparison to T2 or no other psychoactive prescribed drugs than opioid drug at both time points were put in the group of reduced use of psychoactive drugs (42%). The rest were put into group of non-reduced use of psychoactive drugs (58%). Table 3 presents the demographic characteristics of the sample II. In the buprenorphine group, 78% of the patients were given buprenorphine/naloxone at all test points. Table 4 shows other medication characteristics of the sample II within the last 24 h before testing.

**Table 3:** Group demographics in sample II

	Buprenorphine or Buprenorphine/ Naloxone (*n *= 18)	Methadone (*n *= 18)	Group or time point comparison *p*-values
Age, years at T1 (*M ± SD*)	30 ± 8	32 ± 8	*ns*

Sex: females/males, %	28/72%	33/67%	*ns*

Verbal IQ ^a^ (*M ± SD*)	101 ± 8	100 ± 11	*ns *

Education, years (*M ± SD*)	10 ± 2	11 ± 1	*ns*

Participants with early neurobehavioral problems %	33%	28%	*ns*

Examined in inpatient settings %			
T2	6%	6%	*ns*
T3	11%	11%	*ns*

Participants with high frequency use of any substance of abuse % ^b^			
T2	44%	39%	*ns*
T3	44%	44%	*ns*

Participants with recent month extra doses of any opioid % ^c^			
T2	36%	36%	*ns*
T3	36%	43%	*ns*

Nicotine, participants using daily, %			
T2	100%	100%	*ns*
T3	100%	100%	*ns*

Days in opioid substitution treatment at test (*M ± SD*)			
T2	211 ± 19	196 ± 27	*ns*
T3	411 ± 43	405 ± 29	*ns*

Age of onset, any substance abuse (*M ± SD*)	16 ± 4	15 ± 3	*ns*

Age of onset, opioid abuse (*M ± SD*)	19 ± 5	19 ± 4	*ns*

Participants with lifetime alcohol abuse	72%	83%	*ns*

Years of any substance abuse at T1 (*M ± SD*)	15 ± 7	17 ± 7	*ns*

Years of alcohol abuse at T1 (*M ± SD*)	3 ± 4	3 ± 3	*ns^b^*

Years of opioid abuse at T1, years (*M ± SD*)	10 ± 7	12 ± 7	*ns*

^a ^Estimation based on the WAIS-R Vocabulary score.

^b ^High frequency = three or more days a week Alcohol use was considered heavy if it was at least mean weekly 16 portions for females and 24 portions for males. One portion was defined as 12 g of alcohol.

^c ^Non-prescribed doses of opioids during the recent month seen in drugs screens or admitted by the patient.

**Table 4:** Medications given to participants within the last 24 h before testing in sample II

	Buprenorphine or Buprenorphine/ Naloxone (*n *= 18)	Methadone (*n *= 18)	Group or time point comparison *p*-values
Opioid drug, dose (*M *± *SD *(range) )			
T2	22 ± 5 mg ( 10 - 28 mg)	-	T2 vs. T3, *ns*
T3	21 ± 6 mg ( 6 - 30 mg)	-	
T2	-	119. ± 33 mg (80 - 180 mg)	T2 vs. T3, *ns*
T3	-	129 ± 33 mg (75 - 180 mg)	

Participants using BZD medication			
T2/T3	78%/67%	89%/94%	*ns/ns*
BZD dose at T2 (*M *± *SD *(range))	20 ± 16 mg (0 - 60 mg)	21 ± 16 mg (0 - 70 mg)	T2 vs. T3, *ns* *ns*
BZD dose at T3 (*M *± *SD *(range))	16 ± 14 mg (0 - 40 mg)	20 ± 10 mg (0 - 40 mg)	*ns* T2 vs. T3, *ns*

Number of other medications with possible cognitive effects ^a^			
T2/T3 (*M *± *SD *; (range))	1.8 ± 1.1 (0 - 3)	2.2 ± 0.7 (1 -4)	*ns*
	1.9 ± 1.4 (0 - 4)	2.0 ± 1.0 (1 - 4)	*ns *
			T2 vs. T3, *ns*

These included antidepressants, neuroleptics (used with anxiolytic indications), non-benzodiazepine hypnotics, and substance abuse withdrawal symptom or (non-opioid) pain relievers.

C = controls, M = methadone, BN = buprenorphine or buprenorphine/naloxone

> = superior than, *** = statistically significant at level *p *< 0.001. ** = statistically significant at level *p *< 0.01. * = statistically significant at level *p *< 0.05.

## Procedure

Cognitive tests were administered between three to six hours after opioid substitution drug had been given.

**Attention **was assessed by two tests from the Test for Attentional Performance (TAP) [46]. In the Alertness test, the participant was instructed to respond to visual stimuli by pressing a response key as quickly as possible. The stimuli were presented without or with auditory warning signal. The condition without warning signal is a simple reaction time task reflecting tonic alertness. The condition with auditory warning signal reflects both tonic and phasic alertness. In the Go/NoGo test, the participant was instructed to respond only to two out of five alternative stimuli. Thus, selective attention and executive control of action was assessed.

**Working memory **was assessed by two tests. In the Letter-Number Sequencing task from the Wechsler Memory Scale - III the participant was instructed to repeat letters and numbers in specific order [47]. In the Paced Auditory Serial Addition Task ( PASAT) the participant was instructed to add two consecutive numbers from an auditory series of digit [48]. A new digit was presented after every 1.6 seconds. Both tests are thought to tap complex working memory because simultaneous storage and manipulation of the material is needed.

**Verbal memory **was assessed by the Logical Memory from the Wechsler Memory Scale - III. However, only one story was presented. A full description of the tasks is given in our previous report [7].

### Statistical analyses: stability of function

Longitudinal changes in cognitive function were examined by repeated-measures analysis of variance (ANOVA) using general linear model approach. Group was used as between-subjects factor and time as within-subjects factor. Before the analyses normality assumptions of cognitive variables were examined by Shapiro-Wilk's test and homogeneity of variance by Levene's test. The data were also screened for outlying values. On the basis of these procedures, reaction time and the PASAT scores were subjected to log transformations before further analyses, and the Go/NoGo errors were examined by non-parametric Kruskal-Wallis ANOVA. Sphericity assumption was tested by Mauchly's test, and when appropriate, analyses of effects were interpreted using Huynh-Feldt correction. The effects of demographic variables on cognitive performance were tested as covariates. Only significant covariates were retained in the model. Statistically significant between groups effects were followed by planned contrast using healthy controls as a reference group. Significant time effects we examined using repeated contrast (T2 vs. T1 and T3 vs. T2). When a significant group by time interaction effect was noted, it was examined further by combining previous contrasts (healthy control vs. buprenorphine group * T2 vs. T1, healthy control vs. buprenorphine group * T3 vs. T2; and healthy control vs. methadone group, respectively). All statistical analyses were done by SPSS statistical software, version 15.0, with an exception of the effect size calculations. These were done by an effect size calculator provided by Durham University, UK [49]. For the effect size estimation we pooled the samples and corrected the values by Hedge's correction for small sample bias.

### Statistical analyses: correlates of cognitive functioning

Cognitive tests selected for the analyses were the same as in the part I, except that the PASAT was excluded from the second set of analyses. Improvement in the PASAT is shown to be related to practice effect [50]. This makes it problematic to analyze the correlates of this measure in repeated testing. In order to reduce the number of cognitive variables correlations between the variables analyzed, and whenever justified, domain-wise cognitive sum scores for T2 and T3 performances were formed. T2 performance was used as a reference point in T3 summed scores. Analysis of correlation is sensitive for the effects of outliers. Therefore, visual inspections of scatter plots were used to check the linearity of the relationship between variables and the role of possible outliers. Then correlations between cognitive variables we analyzed by the Pearson product moment method. As expected there were high positive correlations between all reaction time measures at both test points (range .52 - .86); whereas correlations between reaction time measures and other cognitive measures ranged from zero to moderate (-.38 as highest). Therefore, a mean composite score called attention performance was calculated after converting the test scores into z-scores. The working memory measure, the Letter-Number Sequencing task, showed only low to moderate correlations with other measures (.38 as highest) and therefore it was not combined with other measures. The verbal memory measures, immediate and delayed recall of the Logical Memory, correlated strongly at both test points (.80 at T2 and .91 at T3). Therefore, a mean sum score called verbal memory was formed after z-score conversion. Then group differences in cognitive function were examined by repeated-measures analysis of variance (ANOVA) using general linear model approach. Group was used as between-subjects factor and time as within-subjects factor. After this all significant or three highest correlates of each cognitive variable were further examined by checking for intercorrelations between these variables and other variables of interest. Also, medication variables were checked for significant intercorrelations. The sample size did not allow for multiple regression analysis. Instead three highest correlations for each cognitive domain were investigated with analyses of semipartial correlations. Correlations between .10 - .19 were considered to show low association and .20 - 29 mild association. Only some of these are reported. Correlations between, .30 - .49 were considered to show moderate association, .50 - 69 substantial and those .70 or above a strong association [51].

## Results

### Stability of cognitive functioning in sample I

**Figure 1:**
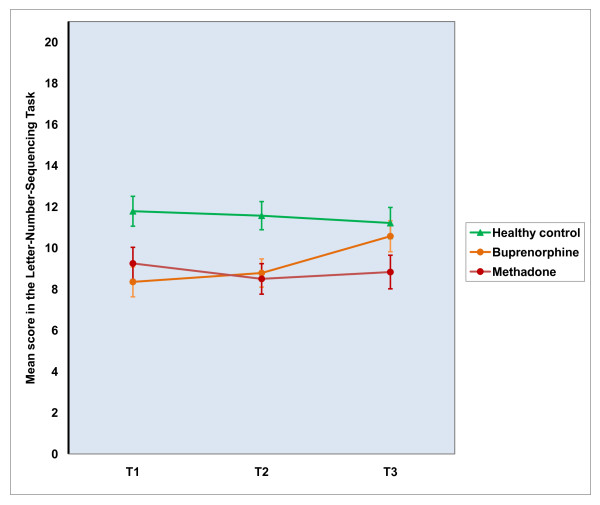
**Group performances in the Letter-Number Sequencing Task during the study period in sample I**.

The pattern of means in Table 5 identifies change over time in cognitive performance in each group. There were statistically significant overall group differences in all attention and memory measures. As apparent from the Table 5, the methadone-treated patient group constantly lagged behind the healthy control group in the TAP reaction time tests measuring alertness and selective attention. Planned contrasts confirmed that the healthy controls outperformed the methadone group in these measures (*p *= 0.002 for the TAP tonic alertness/simple reaction time; *p *= 0.002 for the TAP phasic alertness/reaction time with-auditory-warning-signal; and *p *= 0.001 for the TAP Go/NoGo reaction time/selective attention). There were neither significant time nor group by time interaction effects in these measures. Errors in the Go/NoGo task were rare in all groups, and no significant between groups differences were observed. In both working memory measures there was an overall group effect. In the PASAT the planned contrast revealed that both patient groups performed *overall *worse than the healthy controls at the level of *p *= 0.001. In the Letter-Number Sequencing the values were *p *= 0.016, for healthy controls vs. buprenorphine patients and *p *= 0.008 for healthy controls vs. methadone patients. However, because there was also time effect (the PASAT), or a group by time interaction effect (the Letter-Number Sequencing) in these measures, further analyses are needed before the final interpretation. In the PASAT the improvement in overall performance between T1 and T2 turned out to be non-significant, but the overall improvement between T2 and T3 was significant, *p *= 0.01. As apparent from Figure 1, the source of group by time interaction in the Letter-Number Sequencing was due to differences between the groups between T2 and T3. This was confirmed by a planned contrast which showed improved performance in the buprenorphine patients between T2 and T3 relative to healthy control group, *p *= 0.017. Effect size of the T2 - T3 improvement in the buprenorphine group, as measured by Cohen's **d**, was 0.77. In verbal memory, there was a significant overall group effect both in immediate and delayed condition of the Logical Memory. Both patient groups performed worse than the healthy controls in the immediate Logical Memory, *p *= 0.029 for the buprenorphine group; and *p *= 0.007 for the methadone group. In the delayed Logical Memory the values were *p *= 0.005, and *p *= 0.028, respectively.

**Table 5:** Group comparisons of cognitive performances using repeated measures ANOVA in sample I

TAP Tonic Alertness/simple reaction time (ms)				
T1	232 ± 25	261 ± 21	238 ± 22	Group, *p *= 0.002
T2	236 ± 18	263 ± 21	233 ± 21^a^	Time, *ns*
T3	242 ± 25	267 ± 36	241 ± 25	Group × Time, *ns*
TAP Phasic Alertness/ reaction time with warning signal (ms)				
T1	227 ± 24	244 ± 20	226 ± 21	**Group, *p *= 0.005**
T2	229 ± 21	255 ± 28	224 ± 21 ^a^	Time, *ns*
T3	229 ± 19	254 ± 45	225 ± 22	Group × Time, *ns*

TAP Go-NoGo reaction time (ms)				
T1	490 ± 50	548 ± 74	460 ± 41	**Group, *p *= 0.001**
T2	480 ± 42	548 ± 104	443 ± 72 ^a^	**Group, *p *= 0.002**
T3	493 ± 43	529 ± 63	462 ± 47	**Age, *p *= 0.022**
				Time, *ns*
				Group × Time, *ns*

TAP Go-NoGo errors				
T1	1.1 ± 1.3	0.7 ± 0.6	0.5 ± 0.5	*ns*
T2	0.5 ± 0.7	1.0 ± 0.9	0.5 ± 0.8 ^a^	*ns*
T3	0.6 ± 0.8	0.5 ± 1.0	0.2 ± 0.4	*ns*

The Letter-Number Sequencing				
T1	8.4 ± 2.2	9.3 ± 2.4	11.8 ± 3.4	**Group, *p *= 0.009**
T2	8.8 ± 2.2	8.5 ± 2.3	11.6 ± 3.0	Time, *ns*
T3	10.6 ± 2.2	8.8 ± 2.4	11.2 ± 3.2	**Group × Time, *p *= 0.007**

The PASAT				
T1	32.4 ± 10.5	31.0 ± 8.5	46.3 ± 9.7	**Group, *p *= 0.001**
T2	35.0 ± 6.8	33.4 ± 10.1	45.8 ± 9.0 ^a^	**Time, *p *= 0.013**
T3	35.8 ± 10.0	34.9 ± 11.0	49.8 ± 8.4	Group × Time, *ns*

Logical memory, immediate				
T1	12.8 ± 2.6	14.9 ± 4.5	15.9 ± 3.3	**Group, *p *= 0.016**
T2	13.8 ± 3.1	14.8 ± 3.7	16.3 ± 3.2	Time, *ns*
T3	15.5 ± 4.1	14.3 ± 4.3	17.9 ± 2.9	Group × Time, *ns*

Logical memory, delayed				
T1	11.8 ± 3.0	13.1 ± 4.0	13.9 ± 4.0	**Group, *p *= 0.013**
T2	12.0 ± 4.0	13.7 ± 4.0	15.6 ± 3.1	Time, *ns*
T3	12.4 ± 4.1	11.8 ± 4.7	15.9 ± 3.6	Group × Time, *ns*

Bold indicates statistically significant effects.

^a ^One missing value was replaced by the carry-over value from the preceding testing point.

### Cognitive functioning in sample II

The cognitive group comparisons in the part II (T2 - T3) sample brought results that were in line with the part I sample analyses. Buprenorphine patients outperformed methadone patients in the combined attention performance (*p *= 0.004), and no significant time or group by time effect were seen. In working memory as measured by the Letter-Number Sequencing there was a main effect of time (*p *= 0.01) and a significant group by time interaction, (*p *= 0.04) indicating again that improvement in this measure was due to enhanced performance in the buprenorphine patients between T2 and T3. In the combined verbal memory measure there were no significant differences between groups, time effect, or group by time interaction.

### Correlations between medication variables and non-cognitive variables in sample II

At T2, buprenorphine dose correlated substantially with BZD dose (.62, *p *= 0.006) and moderately with the number of psychoactive drugs (.40, *ns*). In the methadone group, respective values were (.47, *ns; *and .58, *p *= 0.013). At T3, buprenorphine dose correlated at moderate level with BZD dose (.33, *ns*) and at very low level with the number of psychoactive drugs (.10, *ns*). In the methadone group respective values were mild (.25, ns; and .20, ns). In general, buprenorphine or methadone doses did not show significant correlations with substance abuse or demographic variables. As an exception buprenorphine dose correlated negatively with years of alcohol abuse, at T2 the value was -.56 (*p *= 0.016) and at T3 -.64 (*p *= 0.004). In the methadone group, no significant correlations emerged. Other significant correlations between medication variables and other non-cognitive variables of interest are presented in Table 6. It can be noted that high BZD dose was associated with high frequency of substance abuse in the past month and younger age at both time points.

**Table 6:** Significant correlations between medication variables and other non-cognitive variables in sample II

Medication variables	Substance abuse variables	Demographic variables
Benzodiazepine dose (T2)	Frequency of substance abuse in the past month **.36 ( *p *= 0.033)**	Age **-.34 (*p *= 0.040)**

Benzodiazepine dose (T3)	Frequency of substance abuse in the past month **.50 (*p *= 0.002)**	Age **-.33 (*p *= 0.048)**

Number of other psychoactive drugs (T2)		

Number of other psychoactive drugs (T3)	Years of opioid abuse **-.37 (*p *= 0.028)**	

### Correlates of cognitive performances in sample II

**Figure 2:**
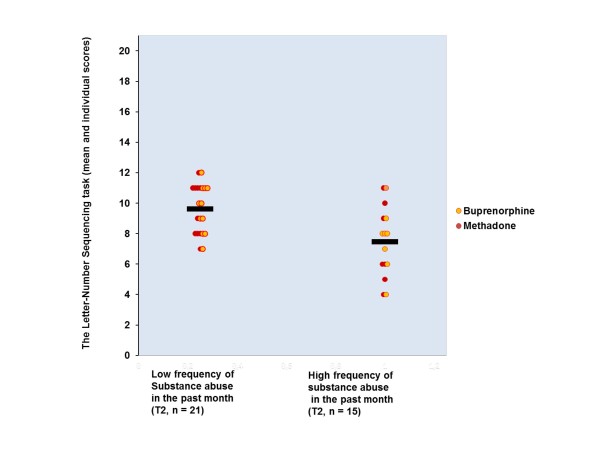
**Correlation between the frequency of the past month substance abuse and the performance in the Letter-Number Sequencing at T2 in sample II**.

**Figure 3:**
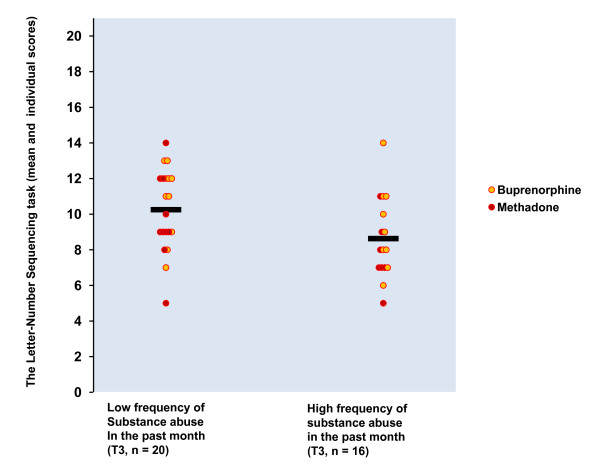
**Correlation between the frequency of the past month substance abuse and the performance in the Letter-Number Sequencing at T3 in sample II**.

As shown in Table 7, the only significant correlate for attention performance at both test points was the opioid substitution drug group. High frequency of substance abuse correlated negatively with the Letter-Number Sequencing performance at both time points. Figures 2 and 3 depict this association. It can be noted from these Figures that the association between working memory performance and frequency of substance abuse in the past month is similar in both groups. The T2 negative correlation remained significant after controlling for two next highest correlates. The T3 correlation dropped to non-significant level after controlling for two next highest correlates (-.18). At T3, high benzodiazepine dose correlated negatively with the Letter-Number Sequencing performance. After controlling for the two other highest correlates, this association was no longer significant (-.22). In further analysis no evidence in support of high association between BZD dose and the Letter-Number Sequencing performance was seen, because T2 correlation between these variables was at zero level (.02). Belonging to the buprenorphine group was the only variable that correlated significantly (.34) with change of the Letter-Number Sequencing performance. After controlling for two other highest correlates, this association was no longer significant.

**Table 7:** Highest correlations between cognitive and non-cognitive variables in sample II

Domain or test	Medication variables	Substance abuse variables	Demographic variables	Significant correlations after controlling for two other correlates
Attention (T2)	Opioid substitution drug .**48 (*p *= 0.003)** Number of other psychoactive drugs (T2) .24	Opioid abuse onset age .25		Opioid substitution drug **.46, (*p *= 0.004)**

Attention (T3)	Opioid substitution drug .**37 (*p *= .024)**	Opioid abuse onset age .28	Age .26	Opioid substitution drug .**37, (*p *= 0.021)**

The Letter-Number Sequencing Task (T2)	Number of other psychoactive drugs .25	Frequency of substance abuse in the past month **-49 (*p*= .002)**	Verbal IQ .29	Frequency of substance abuse in the past month **-44 (*p *= .005)**

The Letter-Number Sequencing Task (T3)	Benzodiazepine dose **.-38**	Frequency of substance abuse in the past month **.-34 (*p *= .044)** Years of opioid abuse .28		

Change score in the Letter-Number Sequencing Task (T3 - T2)	Opioid substitution drug .**34 (*p *= .039)**		Change in the opioid agonist dose -.33 Change in the number of psychoactive drugs -.24	

Verbal memory (T2)	Number of other psychoactive drugs (T2) .25	Frequency of substance abuse in the past month **-. 34 (*p *= .044)**	Verbal IQ .28	

Verbal memory (T3)	Number of other psychoactive drugs (T3) .31		Verbal IQ .32 Years of education .27	Number of other psychoactive drugs **.34 (*p *= .035)**

Bold indicates statistically significant correlation.

The number of psychoactive drugs correlated positively with verbal memory performances at both testing points. At T3, the positive association with the number of psychoactive drugs reached significant level after two other correlates were taken into account. At T2, there was a negative association with the highly frequent past month substance abuse and verbal memory performance. After controlling for two other highest this correlation dropped to non-significant level (.28). Furthermore, at T3 the correlation between highly frequent substance abuse in the past month and verbal memory was very low and to the opposite direction (-.08).

Correlations between opioid substitution drug dose and cognitive performances opioid drug doses could be examined only group-wise (n = 18 in both groups). None of the correlations reached statistical significance. Because there was a significant group by time interaction in the Letter-Number Sequencing indicating specific improvement in this task in the buprenorphine group, correlates for the improvement in the buprenorphine group were examined. No significant correlates for the change score emerged.

## Discussion

This study was designed to evaluate stability and correlates of cognitive functioning in unselected buprenorphine- vs. methadone treated opioid-dependent patients during the first year in OST. The main findings are the following. Buprenorphine-treated opioid-dependent patients do not show deficits in attention, improve in one of the working memory tests, the Letter-Number Sequencing, but they show stable deficits in the other working memory test, the PASAT, and verbal memory. Methadone-treated opioid-dependent patients show stabile cognitive deficits in attention, working memory, and verbal memory. When correlates of cognitive performances are analyzed 6 and 12 after the start of the OST drug type (buprenorphine vs. methadone) is moderately associated with attention performance. Highly frequent substance abuse in the past month is negatively associated with performance in the Letter-Number Sequencing. The number of other psychoactive drugs and verbal IQ both show mild positive correlation with verbal memory.

### Stability of buprenorphine patients' cognitive function during the first year in treatment

Our observation of no reaction time deficits in buprenorphine-treated opioid-dependent patients in relation to healthy controls is in accordance with the idea that some of the negative effects of buprenorphine on cognition disappear after the development of tolerance. Most patients had abused buprenorphine before the treatment (Table 1). Further studies are needed to examine if buprenorphine patients' normal performance in attention tests is related to the development of tolerance only, or if a population selection process is affecting performance in patient samples.

Our finding of partial recovery of working memory function in buprenorphine-treated patients during the OST is in line with the idea of Spiga et al. [52]. The idea is supported by observations by Pirastu et al. showing that buprenorphine patients outperform methadone patients in spatial working memory [18]. They suggest that buprenorphine could preserve working memory function better than methadone because of its antagonism on kappa opioid receptor, which then affects prefrontal dopamine tone known to be important for working memory. This reasoning, however, does not explain why the improvement in working memory in our study took place between 6 and 12 months in the treatment.

In the other working memory measure, the PASAT, both patient groups are inferior to healthy controls while all groups show improvement during the study period. Improvement that is seen in all groups is a normal finding when the PASAT is administered, and most likely reflects practice effect [50]. The result of no specific improvement in the buprenorphine patients in this measure may be related to the finding that also several other cognitive processes than working memory are needed for good performance in the PASAT [53].

In verbal memory buprenorphine-treated patients perform worse than healthy controls during the whole follow-up. Buprenorphine dose given to our patients was relative high (range mean 16 mg (T1) - 21 mg (T3)). High dose of buprenorphine (32 mg) have been associated with verbal memory impairment [54]. In addition, in recent study by Messinis et al. buprenorphine-treated opioid-dependent patients with a fairly low mean dose of buprenorphine (7 mg) performed worse than healthy controls in verbal memory. Abstinent opioid-dependent patients treated with mu opioid antagonist naltrexone showed no significant difference relative to healthy controls. In sum, buprenorphine may negatively affect verbal memory, although evidence is still insufficient.

### Stability of methadone patients' cognitive function during the first year in treatment

In this study, methadone patients show cognitive deficits in all domains studied: attention, working memory and verbal memory. Not all studies, however, have shown attention deficits among them. Gordon found that methadone-treated opioid-dependent patients outperformed controls in simple visual and visual multiple choice reaction times [55]. Curran et al. found that 3 h after methadone dose opioid-dependent patients in methadone-aided opioid withdrawal actually had faster simple reaction times than before the dose [56]. On the other hand, in the Lintzeris et al study high dose of methadone (150% of normal dose) was associated with slower reaction times in OST patients [20]. Thus, the issue whether methadone dose prolongs reaction times in opioid-dependent patients is not fully resolved.

We found a stabile working memory deficit in both complex working memory measures, the Letter-Number Sequencing and the PASAT, in methadone patients. In early study Gritz et al. found no deficit in methadone patients in "simple" working memory test, the Digit Span from the Wechsler scales, in which the items needs to repeated without organizing them [57]. However, in a more recent study Darke et al. found medium effect size difference between methadone patients and healthy controls in the same test [5]. Interestingly, in abstinent opioid-dependent patients "simple" working memory seems to be spared while complex working memory performance is impaired [58,59]. Thus, it would be informative to compare methadone patients against abstinent opioid-dependent patients using both simple and complex working memory measures.

Methadone patients were inferior to healthy controls in verbal memory. Also, in the Darke et al. study opioid-dependent patients treated with methadone for a minimum 5 months were impaired relative to healthy controls in verbal memory [5]. However, in the Curran et al. study opioid-dependent patients treated with methadone for a minimum 6 months were given their normal dose, 33% increased dose, or placebo linctus; and then tested 3-4 after the dose. No significant treatment effect was seen, and the authors conclude that single doses of methadone are devoid of verbal memory effects among long-term methadone users. Thus, negative effect of methadone on verbal memory is not well-confirmed.

### Correlates of cognitive functioning in opioid substitution treated patients

The most consistent finding of analyses of correlates of cognitive functioning after 6 (T2) or 12 months (T3) in treatment is that belonging to the methadone group negatively associates with attention performance. However, as stated earlier in randomized or well-controlled studies methadone patients, in general, have performed at equal level than buprenorphine ones in tests measuring attention. Thus, it is possible that patient selection or other medication or substance abuse factor is affecting the results in non-randomized studies, in which methadone patients perform worse than buprenorphine patients.

We hypothesized that the number of prescribed psychoactive drugs given to the patients would show negative correlations with performance in cognitive tests. Our results, however, show three mild to moderate *positive *correlations between the number of psychoactive drugs and verbal memory. Thus, the results do not confirm the hypothesis that the number of psychoactive drugs as such would correlate negatively with cognitive performance in OST patients. We hypothesized that those with the high opioid substitution drug dose would have higher BZD doses. The results were in line with this hypothesis. Benzodiazepine use was very common in both patient groups, and experimental studies have shown that benzodiazepines, when given in combination with opioid substitution drug may affect negatively attention or verbal memory functioning [24]. Therefore, we hypothesized that a negative correlation between the BZD dose and cognitive measures would be seen. Although one moderate negative correlation between working memory measures and BZD dose is seen in our clinical sample, this does not remain significant when two other correlates are taken into account. In sum, substantial differences between test points and many significant intercorrelations show that relationships between medication variables and cognitive performance are not easily discovered in clinical sample studies.

High frequency of substance abuse in the past month was negatively associated with the working memory measure with executive function component, the Letter-Number Sequencing, at both test points. This finding is line with studies reporting negative association between working memory and recent substance abuse, possibly affecting fluid intelligence in general [31,58,60]. In addition, frequency of substance abuse in the past month correlates positively with BZD dose at both test points (.36 - .50), and BZD dose correlated negatively with the T2 Letter-Number Sequencing performance. Furthermore, the opioid substitution drug doses show moderate or substantial correlations with the BZD doses. There is temptation to suggest an association between the past month frequent substance abuse, high opioid agonist dose, high BZD dose, and impaired working memory performance. Yet, our data do not allow controlling for all these intercorrelations.

The hypothesis of negative effect of lifetime substance abuse on cognitive performance was examined using substance abuse onset ages and durations of abuse as correlates for cognitive performance. Some negative correlations emerged, but these were moderate at best.

Demographic variables have been shown to be important correlates for cognitive performance in opioid-dependent patients [10,36,37]. In our study, the only consistent finding is the positive correlation between Verbal IQ measured by the vocabulary test and verbal memory. This relationship is not surprising because vocabulary and verbal memory correlate moderately in normal and clinical populations [61,62].

### Limitations

The main limitation of part I of this study is the fact that, while the opioid-dependent patient groups were comparable to each other in variables of interest, our healthy comparison group had hardly any medication or substance abuse. Although these differences relate to the 'dark side' of addiction [63] they limit the specificity of our results. Some of the cognitive deficits seen in patients may be premorbid or related to early-onset substance abuse [64,65]. In order to examine these questions analyses of correlations were done in extended population in part II of our study.

Because of high drop-out rate in our study we could not use statistical methods to test causal relationships in part II. On the other hand, comparison of correlations from two testing points gives possibility to evaluate their reliability and consistency. In case of prescription opioid drug, drug screen do not show extra doses. Thus, it is possible that opioid doses are not fully accurate. While much is known about the pharmacological comparisons between different BZDs, the values of BZD equivalent doses are approximations instead of precise values [39]. Alcohol use estimates may not be fully accurate. These estimates were based on information given by the participants. Breath alcohol analyzer or other objective test was used only when considered necessary. Finally, our results do not imply that functional capacity of an opioid-dependent patient could be determined on the basis of his/her drug group. Instead, validation of cognitive test battery to a functional task, for instance driving a car, as well as exploration of non-cognitive factors is needed [66]. Only then individual assessment of the functional capacity can be made.

## Conclusions

In conclusion, our results show again that in non-randomized clinical studies buprenorphine patients tend to perform better than methadone patients. The results do not support the idea that there would be substantial negative associations with medication variables and cognitive performance among patients in OST. A longitudinal study of opioid substitution treated patients who switch from buprenorphine to methadone or vice versa would be ideal in detecting cognitive effects of these drugs and the roles of other clinical variables.

## Competing interests

Pekka Rapeli has given a paid lecture in training organized by Schering-Plough, the former manufacturer of buprenorphine.

## Authors' contributions

PR planned and performed cognitive testing and statistical analysis. He wrote the first version of the manuscript and prepared the final manuscript. HA conceived the idea of the study and advised in manuscript preparation. HK participated in the design of the study and in manuscript preparation. CF carried out psychiatric investigations. All authors prepared, read and accepted the final manuscript.

## Pre-publication history

The pre-publication history for this paper can be accessed here:

http://www.biomedcentral.com/1472-6904/11/13/prepub
